# DNA lipid nanoparticle vaccine targeting outer surface protein C affords protection against homologous *Borrelia burgdorferi* needle challenge in mice

**DOI:** 10.3389/fimmu.2023.1020134

**Published:** 2023-03-16

**Authors:** Annabelle Pfeifle, Sathya N. Thulasi Raman, Casey Lansdell, Wanyue Zhang, Levi Tamming, Jonathon Cecillon, Emmanuel Laryea, Devina Patel, Jianguo Wu, Caroline Gravel, Grant Frahm, Jun Gao, Wangxue Chen, George Chaconas, Simon Sauve, Michael Rosu-Myles, Lisheng Wang, Michael J. W. Johnston, Xuguang Li

**Affiliations:** ^1^ Centre for Oncology, Radiopharmaceuticals and Research, Biologic and Radiopharmaceutical Drugs Directorate, Health Products and Food Branch, Health Canada and World Health Organization Collaborating Center for Standardization and Evaluation of Biologicals, Ottawa, ON, Canada; ^2^ Department of Biochemistry, Microbiology and Immunology, Faculty of Medicine, University of Ottawa, Ottawa, ON, Canada; ^3^ Department of Chemistry and Biomolecular Sciences, Faculty of Science, University of Ottawa, Ottawa, ON, Canada; ^4^ Centre for Vaccines, Clinical Trials and Biostatistics, Biologic and Radiopharmaceutical Drugs Directorate, Health Products and Food Branch, Health Canada and World Health Organization Collaborating Center for Standardization and Evaluation of Biologicals, Ottawa, ON, Canada; ^5^ Human Health Therapeutics Research Center, National Research Council of Canada, Ottawa, ON, Canada; ^6^ Department of Biochemistry and Molecular Biology and Department of Microbiology, Immunology and Infectious Diseases, Cumming School of Medicine, Snyder Institute for Chronic Diseases, University of Calgary, Calgary, AB, Canada; ^7^ Department of Chemistry, Carleton University, Ottawa, ON, Canada

**Keywords:** Lyme disease, lipid nanoparticle, outer surface protein C, antibodies, carditis, lymphadenopathy, DNA vaccine, Lyme borreliosis

## Abstract

**Introduction:**

The incidence of Lyme disease (LD) in Canada and the United States has risen over the last decade, nearing 480,000 cases each year. *Borrelia burgdorferi* sensu lato, the causative agent of LD, is transmitted to humans through the bite of an infected tick, resulting in flu-like symptoms and often a characteristic bull’s-eye rash. In more severe cases, disseminated bacterial infection can cause arthritis, carditis and neurological impairments. Currently, no vaccine is available for the prevention of LD in humans.

**Methods:**

In this study, we developed a lipid nanoparticle (LNP)-encapsulated DNA vaccine encoding outer surface protein C type A (OspC-type A) of *B. burgdorferi.*

**Results:**

Vaccination of C3H/HeN mice with two doses of the candidate vaccine induced significant OspC-type A-specific antibody titres and borreliacidal activity. Analysis of the bacterial burden following needle challenge with *B. burgdorferi* (OspC-type A) revealed that the candidate vaccine afforded effective protection against homologous infection across a range of susceptible tissues. Notably, vaccinated mice were protected against carditis and lymphadenopathy associated with Lyme borreliosis.

**Discussion:**

Overall, the results of this study provide support for the use of a DNA-LNP platform for the development of LD vaccines.

## 1 Introduction

Nearly half a million people in North America are estimated to develop Lyme disease (LD) each year (1, 2). Since the implementation of standardized surveillance, the number of reported cases has increased 2- and 20-fold in the United States and Canada, respectively (2, 3). Lyme disease is caused by bacteria of the *Borrelia burgdorferi* sensu lato complex, which are transmitted to humans *via* ticks of the *Ixodes* genus. Infection with *B. burgdorferi* often results in *erythema migrans*, more commonly known as a “bull’s-eye” rash, and flu-like symptoms. Moreover, bacterial dissemination can lead to more severe clinical manifestations such as arthritis, carditis, and neurological impairments (4–6). Given the current challenges in diagnosing and treating LD, novel prevention strategies are critical in reducing the LD health care burden.

Currently, there is no vaccine available for the prevention of LD in humans. The development of LD vaccines has primarily focused on two immunogenic lipoproteins located on the surface of *B. burgdorferi*, outer surface protein A (OspA) and outer surface protein C (OspC). OspA is responsible for colonization of the tick vector, while OspC is required to initiate and maintain early infection in the host (7, 8). OspA and OspC are reciprocally expressed throughout the tick feeding cycle, with expression of OspA highest while the bacteria reside in the tick midgut (9). Following a blood meal, the spirochete downregulates OspA expression to facilitate migration into the salivary glands and expression remains low throughout infection of the mammalian host (10, 11). Consequently, to prevent transmission during feeding, vaccines targeting OspA must induce high titres of antibodies in the host prior to encountering the pathogen. Furthermore, vaccines targeting OspA are thought to require frequent boosters to maintain high circulating antibody titres (12). In contrast, OspC is highly expressed by the bacteria in response to hematophagy and during initial infection of the mammalian host (9, 10). Importantly, antibodies targeting both OspC and OspA are capable of borreliacidal activity, an important mechanism of spirochete clearance (13–15). However, only OspC-containing vaccines may confer host-based protection. While some licensed veterinary vaccines contain OspC antigens in addition to OspA, there are currently no approved vaccines that target OspC alone.

The majority of past LD vaccine research has centered on recombinant subunit vaccines. Next-generation vaccine platforms, such as DNA vaccines, offer clear advantages over protein subunit vaccines, including improved cost-efficiency and ease of production (16, 17). Previous studies investigating the use of DNA vaccines for the delivery of OspA or OspC antigens, have demonstrated strong immunogenicity and protection against challenge (18–22). However, these studies employed the use of gene guns, tail-tattoo, or intradermal injection for DNA delivery. Despite the observed efficacy, these administration techniques may not be easily translated to human vaccination programs. In comparison, liposomes and lipid nanoparticles have been shown to be a safe and reliable method of delivering DNA by intramuscular injection (23–25). While these particles have been explored in detail for mRNA delivery, including their use in the Comirnaty (Pfizer-BioNTech) and Spikevax (Moderna) COVID-19 vaccines, further research is required to evaluate their potential for DNA-based vaccination against a breadth of infectious agents (26, 27). In this study, we present a plasmid DNA vaccine encoding OspC-type A encapsulated in a lipid nanoparticle (pVAX1-OspC:LNP). Following the injection of two doses of the candidate or control vaccine in mice, we evaluated the serum antibody response and corresponding *in vitro* borreliacidal activity. We also used qPCR and culture microscopy to assess the bacterial burden following challenge with *B. burgdorferi* expressing OspC-type A. Finally, histopathology was performed to observe the development of carditis and lymphadenopathy following challenge.

## 2 Materials and methods

### 2.1 Bacterial cell culture

*Borrelia burgdorferi* strain B31-A3 used for challenge and strain B31-A3 constitutively expressing OspC-type A (*B. burgdorferi-*OspC-con) used for the borreliacidal assay were kindly provided by Dr. Patricia Rosa (National Institute of Allergy and Infectious Disease, Montana, US). The derivation of strain B31-A3 from strain B31 MI and engineering of *B. burgdorferi*-OspC-con were previously described (28, 29). *B. burgdorferi* strains were cultured from glycerol stocks in complete Barbour-Stonner-Kelly (BSK)-H media containing 6% rabbit serum (Millipore Sigma, Burlington, ON) and supplemented with 5 µg/mL Amphotericin B, 100 µg/mL Phosphomycin, and 50 µg/mL Rifampicin. Cultures were incubated at 35°C and 1.5% CO_2_ until mid-log phase and were not sub-cultured. Enumeration was performed using a Petroff-Hausser counting chamber under dark-field microscopy. Plasmid content of the wild-type B31-A3 strain was validated by multiplex PCR and OspC expression was confirmed by the induction of OspC-specific anti-sera following infection in naïve mice.

### 2.2 Animal care

Six-week old female C3H/HeN mice were obtained from Charles River, Senneville, Quebec, Canada. All animal procedures were approved by the Animal Care Committee in Health Canada, Ottawa and performed in accordance with institutional guidelines.

### 2.3 DNA vaccine generation

The type A outer surface protein C (OspC) gene of *Borrelia burgdorferi* B31 (GenBank accession #AAC66329.1) was designed to include the human tyrosinase signal peptide (MLLAVLYCLLWSFQTSAGHFPRA; GenBank accession #AH003020) at the N-terminus (30, 31). The coding region was optimized for expression in mice and commercially synthesized in a pUC57 plasmid vector by Bio Basic Inc. (Markham, ON). The OspC gene containing the signal sequence was sub-cloned into a pVAX1 plasmid vector (ThermoFisher, Ottawa, ON), using NotI-HF and EcoRI-HF restriction enzymes (New England Biolabs, Whitby, ON). Large-scale amplifications of the pVAX1-OspC plasmid were generated using the QIAGEN Plasmid Giga Kit (Montreal, QC) according to the manufacturer’s instructions. The genetic sequences were validated by Sanger Sequencing prior to nanoparticle encapsulation.

### 2.4 Lipid nanoparticle (LNP) synthesis

LNPs were synthesized by rapid microfluidic mixing of an aqueous phase containing the plasmid DNA with an ethanol phase containing the lipids. Briefly, the aqueous phase was prepared in 25 mM acetate buffer, pH 4, containing pVAX1 or pVAX1-OspC-type A DNA. The ethanol phase was comprised of ionizable lipid: phospholipid: cholesterol: PEG-lipid at a ratio of 50: 10: 38.5: 1.5 mole %, respectively. Lipids used were 2,2-dilinoleyl-4-dimethylaminoethyl-[1,3]-dioxolane (DLin-KC2-DMA, MedKoo Biosciences, Inc., Morrisville, North Carolina, USA), 1,2-dioleoyl-sn-glycero-3-phosphocholine (DOPC, Millipore Sigma), ovine cholesterol (Cholesterol, Millipore Sigma), and 1,2-dimyristoyl-rac-glycero-3-methoxypolyethylene glycol-2000 (DMG-PEG2000, Millipore Sigma). The two phases were mixed using a NanoAssemblr BT™ instrument (Precision Nanosystems, Inc., Vancouver, BC) with a microfluidics cartridge containing a staggered herringbone mixing unit (Total Volume 1.5 mL; Flow Rate Ratio 3:1 aqueous:organic; Total Flow Rate 5 mL/min). The resulting LNPs were dialysed against phosphate-buffered saline (PBS, ThermoFisher) for 18 h at 4°C in a 10k MWCO cassette (ThermoFisher), then concentrated using an Amicon Ultra 4 10k MWCO centrifugal concentrator (Millipore Sigma). For both the prime and boost vaccinations, fresh batches of DNA-LNPs were prepared within three days of injection and stored at 4°C.

### 2.5 LNP characterization

Nanoparticle size was measured in PBS by nanoparticle tracking analysis (NTA) (NanoSight, Malvern Panalytical, Westborough, MA, USA) under static conditions. For each sample, a 1.2 mL dilution was prepared in PBS and five consecutive tracking videos (1 min each) were recorded, with the sample syringe being advanced by 100 µL between each video. NTA sizing data is the result of merging the five separate tracking videos (5 x 1 min videos).

Nucleic acid encapsulation efficiency in LNP samples was measured using Triton X-100 (Millipore Sigma) and SYBR™ Gold (Thermo Fisher). Briefly, LNPs were disrupted with 1% Triton X-100 in PBS in a 96-well plate. 1X SYBR™ Gold dye in PBS was added to the sample and the plate was read using a Synergy MX plate reader (Ex/Em: 495/537 nm) (BioTek, Winooski, Vermont, USA). Total nucleic acid in the sample was determined by applying the relative fluorescent units to a standard curve of nucleic acids with detergent. Non-disrupted LNPs were also measured concurrently under the same conditions, but without detergent, to detect un-encapsulated nucleic acid. The amount of DNA encapsulated in LNPs was determined by subtracting the un-encapsulated DNA from the total amount of DNA in a sample. Finally, the amount of encapsulated DNA was divided by the total amount of DNA in a sample to obtain the encapsulation efficiency.

### 2.6 Immunizations and bacterial challenge

Groups of five eight-week old mice were injected intramuscularly into the tibialis anterior with lipid nanoparticles containing 12 µg of pVAX1 (pVAX1:LNP) or pVAX1-OspC-type A (pVAX1-OspC:LNP). The injection volume was 50 µL distributed between two injections sites, one in each leg. A second identical vaccination was administered four weeks after the initial injection. Four weeks after the boost injection, mice were challenged subcutaneously between the shoulder blades with 1 × 10^5^ *B. burgdorferi*. For challenge, wild-type *B. burgdorferi* B31-A3 was grown to mid-log phase in complete BSK-H media as described above. Cultures were centrifuged at 5000 × g for 20 minutes and resuspended in incomplete BSK-H media without rabbit serum (Millipore Sigma). Cells were counted by dark-field microscopy and diluted to a final concentration of 1 × 10^6^ cells/mL. Finally, mice were sacrificed 14 days after challenge for the collection of blood and tissues.

This study was repeated once with an additional three mice per group following the same above-mentioned procedure. The data from both studies were combined for a total of eight mice per group, represented in each figure.

### 2.7 Enzyme linked immunosorbent assay

96-well Nunc Maxisorp™ flat bottom plates (ThermoFisher) were coated with 0.5 µg/mL of recombinant OspC-type A (Native Antigen, Oxfordshire, UK) in carbonate buffer, pH 9.6. Plates were incubated overnight at 4°C then washed three times with PBS containing 0.05% Tween-20 (PBS-T) prior to blocking with 1% (w/v) Bovine Serum Albumin (IgG-Free, Protease-Free) (Jackson Immuno Research, West Grove, PA). For total IgG analysis, two-fold serial dilutions of mouse serum ranging from 1:50 to 1:102,400 were added to the wells and plates were incubated for 1 h at 37°C. For IgG2a and IgG1 analysis, serum was diluted three-fold between the range of 1:100 to 1:17,714,700. Subsequently, plates were washed six times with PBS-T and incubated with HRP-conjugated goat anti-mouse IgG (Cytiva, Marlborough, MA), HRP-conjugated goat anti-mouse IgG1 (Jackson Immuno Research), or HRP-conjugated goat anti-mouse IgG2a (Jackson Immuno Research) diluted 1:5,000 in blocking buffer for 1 h at 37°C. The plates were washed six times with PBS-T prior to the addition of 100 µL of Tetramethylbenzidine (TMB) substrate (Cell Signaling Technology, Danvers, MA). After incubating for five minutes at room temperature, the reaction was terminated with 0.16 M sulfuric acid. Finally, the absorbance at 450 nm was measured using a spectrophotometer. Endpoint titres were defined as the reciprocal of the highest dilution that resulted in an OD greater than the average OD of all wells containing serum from mice vaccinated with the pVAX1:LNP vaccine plus three times the standard deviation.

### 2.8 Serum borreliacidal assay

Cultures of *B. burgdorferi* B31-A3 constitutively expressing OspC type A (*B. burgdorferi*-OspC-con) were grown to mid-log phase and diluted to 5 × 10^7^ cells/mL. Four microlitres of culture were then added to 0.2 mL microtubes, followed by 4 µL of mouse serum and 4 µL of guinea pig serum (GPS, Complement Technologies, Tyler, TX). Reaction volumes were topped to 20 µL with complete BSK-H media and incubated at 35°C and 1.5% CO_2_ for 24 hours. Motile cells were counted using a Petroff-Hausser counting chamber under dark-field microscopy. A negative control consisting of serum from a naïve mouse heat-inactivated for 56°C for 30 min was also included. Percent live spirochetes was determined by dividing the cell count by the average cell count of negative control samples.

### 2.9 qPCR for bacterial burden

At necropsy, the bladder, left ear, left tibiotarsal joint, inferior half of the heart, and left popliteal and medial iliac lymph nodes were collected and frozen in liquid nitrogen. DNA was extracted from tissues using a DNeasy Blood & Tissue Kit (Qiagen) according to the manufacturer’s instructions. DNA samples were diluted to match the lowest concentration sample and 5 µL of DNA was added to each well of a 96-well MicroAmp™ Fast Optical 96-Well Reaction Plate (ThermoFisher) containing 10 µL TaqMan Fast Advanced MasterMix and 1 µL of either *Borrelia burgdorferi FlaB* custom gene assay (Forward primer: 5’-TCTTTTCTCTGGTGAGGGAGCT-3’, Reverse primer: 5’-TCCTTCCTGTTGAACACCCTCT-3’, ThermoFisher) or mouse β-actin gene assay (ID: Mm02619580_g1, ThermoFisher). Reaction volumes were topped to 20 µL with nuclease-free water. qPCR reactions were performed using an Applied Biosystems™ 7500 Fast Real-time PCR instrument. Standard curves of plasmids encoding mouse β-actin or extracted *B. burgdorferi* genomic DNA were used to determine the number of FlaB copies per 1 × 10^6^ β-actin copies.

### 2.10 Tissue culture for bacterial identification

At necropsy, the right ear and skin surrounding the bacterial injection site were collected and placed separately in complete BSK-H media containing 6% rabbit serum and supplemented with 5 µg/mL Amphotericin B, 100 µg/mL Phosphomycin, and 50 µg/mL Rifampicin. Cultures were incubated for fourteen days at 35°C and 1.5% CO_2_ then examined by darkfield microscopy. Cultures were considered positive if at least one spirochete was identified across five fields of view.

### 2.11 Determination of lymphadenopathy

The left and right axillary lymph nodes were collected at necropsy and placed together in cold RPMI media supplemented with 0.15% (w/v) sodium bicarbonate, 1 mM sodium pyruvate, 5 mM HEPES, 20 U/mL Penicillin, 0.02 mg/mL Streptomycin, 0.1% (v/v) 2-mercaptoethanol, and 10% (v/v) heat-inactivated fetal-bovine serum (FBS). Single-cell suspensions were generated by gently pressing lymph nodes between two frosted microscope slides then filtering through a 70 µm cell strainer. Cell suspensions were centrifuged at 300 × g for 5 minutes and resuspended in media. White blood cell counts were obtained using the Sysmex XT-2000iV hematology analyser and represent the counts for a combined suspension of the left and right axillary lymph nodes.

### 2.12 Histopathology

At necropsy, the heart was bisected horizontally and the superior half of the heart, including the origin of the great vessels, heartbase, and top halves of the ventricles and atria was collected. The tissue was immersed in 10% neutral buffered formalin for 48 h before proceeding with trimming and routine histologic processing. Tissues were embedded in paraffin and 4 µm thick sections were stained with Harris hematoxylin (ThermoFisher) and Instant eosin (ThermoFisher) in an Epredia™ Gemini™ AS Automated Slide Stainer (ThermoFisher). Inflammation in the heart sections was subjectively assessed by a certified veterinary pathologist and assigned a numeric grade (0–5) where 0 is normal, 1 is minimal, 2 is mild, 3 is moderate, 4 is marked, and 5 is severe.

### 2.13 Statistical analysis

Statistical significance was calculated using a one-sided Wilcoxon rank-sum exact test at a 5% significance value. All statistical analyses were performed using SAS Enterprise Guide 7.1. * p-value < 0.05, ** p-value < 0.01, *** p-value < 0.001, **** p-value < 0.0001.

## 3 Results

### 3.1 DNA vaccine design and encapsulation

First, we engineered a pVAX1 vector to express the outer surface protein C (OspC) of *Borrelia burgdorferi* B31. To promote antigen secretion, a human tyrosinase signal sequence was added to the N-terminus of the OspC gene (**Figure S1A**). Next, the pVAX1-OspC plasmid was encapsulated into lipid nanoparticles (pVAX1-OspC:LNP) prior to immunization of mice to facilitate nucleic acid delivery. The pVAX1 plasmid, which does not express an antigen of interest, was also encapsulated in LNPs as a negative control (pVAX1:LNP). When analyzed by nanoparticle tracking analysis, both particle formulations resulted in similar size distributions and encapsulation efficiencies (**Table 1**; **Figure S1B**).

**Table 1:** Characterization of DNA-LNP vaccine formulations.

Vaccine Group	Dose	Mean ± SD (nm)	Encapsulation Efficiency
pVAX1:LNP	Prime	93 ± 30	>97%
pVAX1:LNP	Boost	107 ± 38	>99%
pVAX1-OspC:LNP	Prime	92 ± 27	>97%
pVAX1-OspC:LNP	Boost	103 ± 36	>99%

Mean sizes were measured by nanoparticle tracking analysis (NTA). Encapsulation efficiency was determined by SYBR™ Gold assay. SD, standard deviation.

### 3.2 pVAX1-OspC:LNP induces robust antibody response with borreliacidal activity

**Figure 1:**
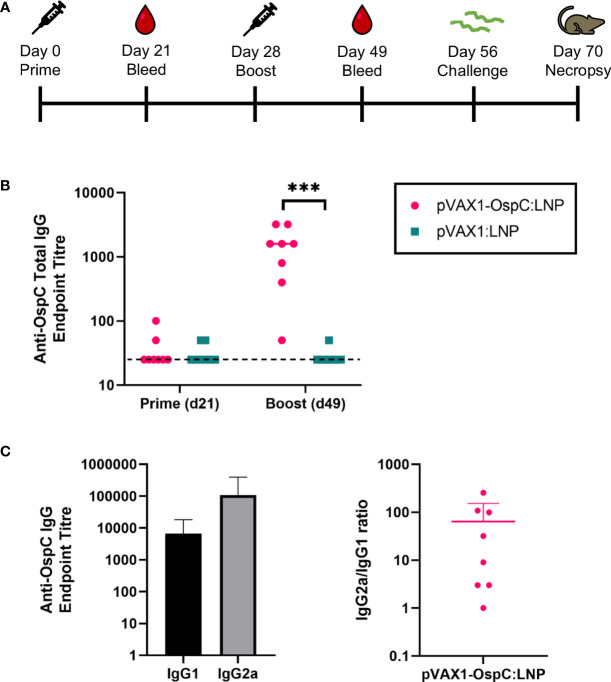
pVAX1-OspC:LNP vaccination is immunogenic in mice. **(A)** Schematic depicting the immunization and challenge schedule in C3H/HeN mice. Mice were bled on day 21 and day 49. Eight mice per group were injected intramuscularly with pVAX1-OspC:LNP or pVax:LNP on day 0 and day 28. Mice were challenged subcutaneously with 1 × 10^5^ *Borrelia burgdorferi* B31 on day 56 and sacrificed on day 70 for tissue collection. **(B)** ELISA determination of OspC-specific IgG antibody titres in serum of mice three weeks after prime (day 21) or boost (day 49) injections. Dashed line indicates the limit of detection. Samples pertaining to data points on the dashed line have antibody titres below the limit of detection. **(C)** Titres and ratio of IgG2a and IgG1 OspC-specific serum antibodies three weeks after boost immunization (day 49). Error bars represent standard deviation. *** p-value < 0.001.

To evaluate the immunogenicity and protection conferred by our candidate vaccine, C3H/HeN mice were injected intramuscularly with 12 µg of either pVAX1-OspC:LNP or pVAX1:LNP (**Figure 1A**). A second dose of the same construct was administered 28 days after the initial prime injection. Three weeks after the prime injection, OspC-type A-specific antibodies were undetectable in the serum of either vaccine group, however, significant anti-OspC-type A antibodies were detected in mice vaccinated with pVAX1-OspC:LNP three weeks after the boost immunization (**Figure 1B**). Analysis of the IgG2a/IgG1 antibody ratio revealed that the antibody response to pVAX1-OspC:LNP vaccination was predominated by the IgG2a subtype, indicating a Th1 immune bias (**Figure 1C**).

**Figure 2:**
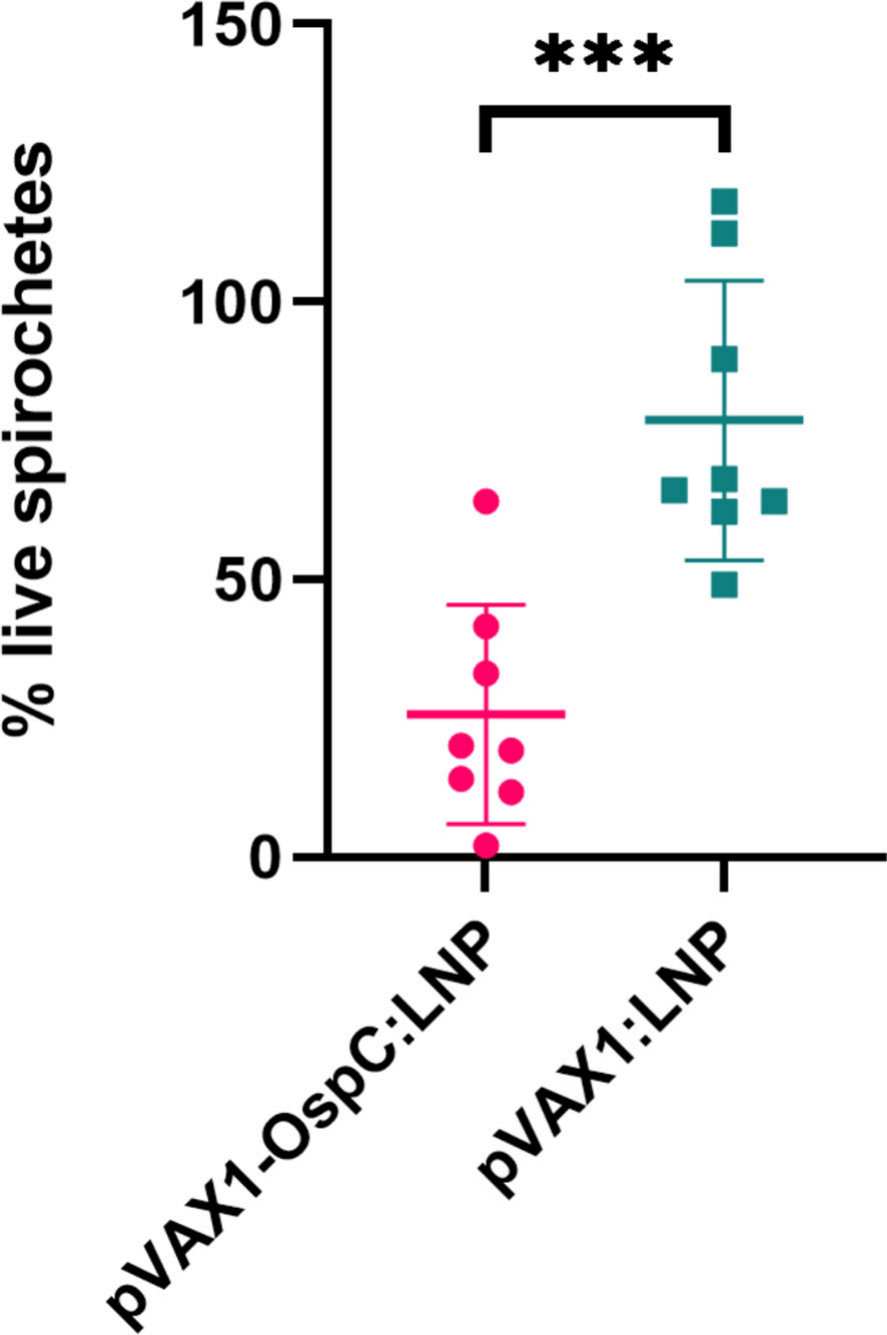
pVAX1-OspC:LNP vaccination generates borreliacidal antibodies. Serum from vaccinated or naïve mice was collected three weeks after boost injection (day 49) and incubated with *B. burgdorferi*-OspC-con for 24 hours. Motile *B. burgdorferi* cells were enumerated by dark field microscopy. Percentage live spirochetes represents the percentage of motile cells compared to naive serum control. Error bars represent standard deviation. *** p-value < 0.001.

Next, we evaluated the bactericidal ability of the antibodies generated in response to vaccination. Investigating the mechanisms of OspC-based vaccine protection can be made difficult by the variability of OspC expression during *in vitro* culture. To best reflect the high OspC expression levels that occur *in vivo*, we conducted the *in vitro* borreliacidal assay using a *B. burgdorferi* stain engineered to constitutively express OspC-type A (*B. burgdorferi-*OspC-con). The bacteria was cultured with mouse serum collected three weeks after boost vaccination in the presence of guinea pig serum for 24 hours. When standardized to a naïve serum control, serum from mice vaccinated with the candidate pVAX1-OspC:LNP vaccine resulted in a 80% reduction in live spirochetes on average (**Figure 2**). In comparison, the serum from mice in the pVAX1:LNP group resulted in an average of only 33% fewer live spirochetes. These results indicate that the candidate vaccine generates functional OspC-specific antibodies that are capable of lysing the spirochete.

### 3.3 pVAX1-OspC:LNP confers protection against homologous *B. burgdorferi* challenge

**Figure 3:**
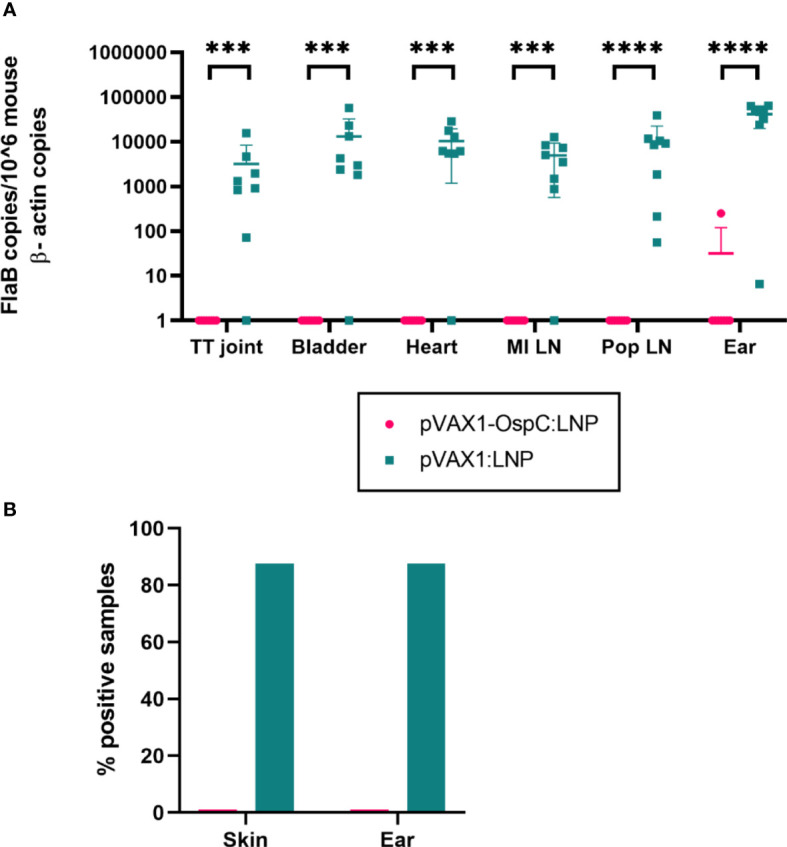
Vaccination with pVAX1-OspC prevents bacterial infection and dissemination. **(A)** qPCR was used to determine bacterial burden in a variety of mouse tissues 14 days after *B. burgdorferi* challenge. Bacterial burden is represented by copies of the *B. burgdorferi* FlaB gene per 1 × 10^6^ mouse β-actin gene copies. Samples below the threshold of amplification were assigned a value of one. TT joint, Tibiotarsal joint; MI LN, medial iliac lymph node; Pop LN, popliteal lymph node. **(B)** The right ear and skin surrounding the bacterial injection site were collected at necropsy, placed in BSK-H growth media, and incubated for 14 days. Cultures were considered positive if at least one spirochete was identified by dark-field microscopy across five fields of view. Error bars represent standard deviation. *** p-value < 0.001, **** p-value < 0.0001.

To evaluate the protection conferred by the candidate vaccine, immunized mice were challenged with 1 × 10^5^ *B. burgdorferi* B31-A3 four weeks after boost vaccination. Two weeks post-challenge, the mice were sacrificed and several tissues were collected to determine the bacterial burden. qPCR analysis of these organs revealed that vaccination with pVAX1-OspC:LNP resulted in *B. burgdorferi* DNA loads below the limit of detection in all tested organs (**Figure 3A**). In comparison, the challenge of mice vaccinated with the control pVAX1:LNP vaccine resulted in significant bacterial burden across all organs. Similarly, cultures of the ears and injection site skin of seven out of eight mice vaccinated with the control pVAX1:LNP were positive for *B. burgdorferi* when examined under dark-field microscopy (**Figure 3B**). In line with the qPCR results, all cultures of the skin and ears obtained from pVAX1-OspC:LNP vaccinated mice were negative for spirochetes when examined by dark-field microscopy. Together, these results demonstrate that vaccination with pVAX1-OspC:LNP fully prevented the establishment and dissemination of *B. burgdorferi* infection in C3H/HeN mice.

### 3.4 pVAX1-OspC:LNP protects against carditis and lymphadenopathy

**Figure 4:**
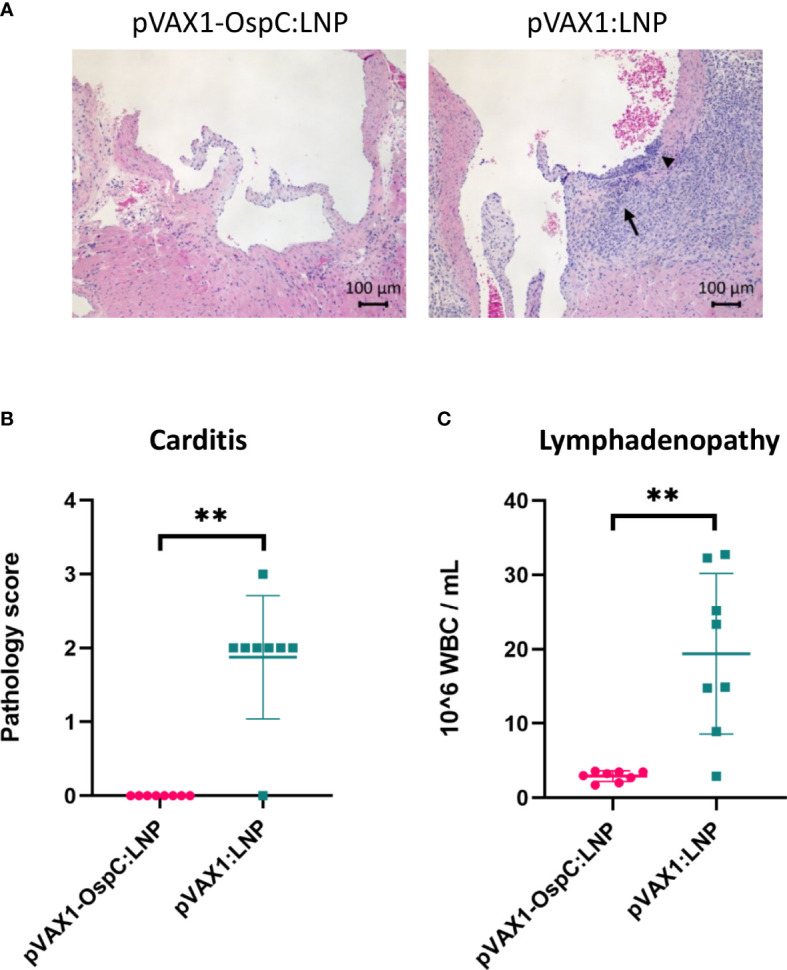
Vaccination with pVAX1-OspC:LNP prevents the development of carditis and lymphadenopathy. **(A)** Representative images of H&E stained heart tissue from mice vaccinated with pVAX1-OspC:LNP or pVAX1:LNP post-challenge. Arrows indicate moderate mononuclear-neutrophilic inflammation in the heart base (long arrow) and transmurally in the aortic origin (short arrowhead). **(B)** Summary of carditis pathology scores as determined by trained histopathologist. Scoring criteria is described in full in the methods section of this article. **(C)** White blood cell (WBC) counts from combined left and right axillary lymph nodes extracted from vaccinated mice two weeks after bacterial challenge. Error bars represent standard deviation. ** p-value < 0.01.

Disseminated infection with *B. burgdorferi* can lead to carditis in both humans and C3H/HeN mice (6, 32, 33). To evaluate the inflammation resulting from *B. burgdorferi* challenge, histopathology analysis of the heart was performed following necropsy. As expected, mice vaccinated with the control pVAX1:LNP vaccine consistently developed mild-to-moderate mononuclear-neutrophilic inflammation of the heart base and aorta 14 days post-challenge (**Figures 4A, B**). In comparison, mice vaccinated with pVAX1-OspC:LNP were protected from developing any detectable carditis. These results are in line with our demonstration that the candidate vaccine prevents bacterial dissemination into the heart tissue.

Acute infection with *B. burgdorferi* can also result in systemic lymph node enlargement, termed lymphadenopathy (34–36). The most severe lymphadenopathy is typically observed nearest to the site of infection (35, 36). For this reason, we collected the axillary lymph nodes at necropsy for enumeration of leukocytes. Control mice that were positive for *B. burgdorferi* infection based on the culture data above had on average eight-fold higher leukocyte counts in the lymph nodes than all *B. burgdorferi*-negative mice, including all mice vaccinated with pVAX1-OspC:LNP (**Figure 4C**). Taken together, these results demonstrate that the candidate pVAX1-OspC:LNP vaccine protects against the development of lymphadenopathy and carditis associated with Lyme disease and bacterial dissemination.

## 4 Discussion

OspC, an immunogenic lipoprotein found on the surface of *B. burgdorferi*, is an attractive target for the development of second-generation LD vaccines. Previous and current LD vaccines in clinical trials have focused on targeting OspA, however, OspC-based vaccines may offer a key advantage. Specifically, expression of OspC is induced by tick feeding and remains high during early infection of the host (9, 10). OspC expression persists for 1-3 weeks in mammals, allowing time for the secondary immune response to facilitate clearance of the infection (37–39) Therefore, unlike vaccines that target OspA and only provide vector-based immunity, OspC-based vaccines have the potential to provide host-based immunity.

When vaccines targeting OspC alone have been evaluated in preclinical trials, variable results have been reported. Wallich et al. and Xiao et al. both found that OspC was not immunogenic in mice when employing a DNA and viral-vector platform, respectively (40, 41). Additionally, oral vaccination with rOspC was found to be immunogenic but not protective against homologous or heterologous tick-challenge (42). In comparison, Scheiblhofer et al. and Klouwens et al. both demonstrated the immunogenicity and protective efficacy of OspC DNA vaccines delivered by gene gun, intradermal injection, or tail-tattoo (19, 22). It should be noted that due to the genetic diversity of the OspC protein both of these studies demonstrated protection only against homologous bacterial strains administered *via* needle- and tick-challenge, respectively. To our knowledge, no OspC-based vaccine has conferred protection against heterologous tick- or needle-challenge to date.

In this report, we demonstrate that vaccination of C3H/HeN mice with a DNA-LNP vaccine targeting OspC-type A afforded effective protection against homologous needle challenge with *B. burgdorferi* B31 (OspC-type A) and the resulting pathology associated with Lyme borreliosis. First, candidate vaccine formulations for prime and boost injections were determined to have mean particle sizes of 92 nm and 103 nm with standard deviations of 27 nm and 36 nm, respectively. These size distributions are within the optimal nanoparticle size range of 20-200 nm for uptake by antigen presenting cells and induction of robust immunogenicity (43–45). In addition, all particle formulations had encapsulation efficiencies >97%, which enables lower cost production at larger scales.

Second, we demonstrated that two doses of the pVAX1-OspC:LNP vaccine induced significant OspC-specific antibodies predominated by a Th1 bias. This finding is in line with that of Wagemakers and colleagues, who reported that vaccination with their DNA-based OspC vaccine by tail-tattoo induced an IgG2a-biased antibody response (20). Additionally, a study by Scheiblhofer et al. suggested IgG1 antibodies may contribute more significantly to vaccine-induced protection, illustrating the need for further studies on the roles of IgG subtypes in the context of LD vaccination and infection (19). In this study, we also demonstrate that antibodies generated in response to the candidate vaccine possess notable borreliacidal activity against a homologous bacterial strain *in vitro*. Clearance of *B. burgdorferi* is known to be largely mediated by the humoral immune response, specifically *via* the classical complement pathway and antibody-dependent cellular phagocytosis (46, 47). Mice deficient in the α-chain of C1q, a component of the classical complement pathway, have demonstrated higher bacterial burdens and dissemination (46). The induction of bactericidal antibodies is therefore a critical feature of LD vaccine development.

Finally, we performed qPCR and microscopy to determine the bacterial burden in a range of tissues following homologous bacterial challenge. All tissue samples from pVAX1-OspC:LNP vaccinated mice were found to be negative for *B. burgdorferi*, indicating that just two doses of the candidate vaccine provided protection from infection and bacterial dissemination. In comparison, previous LD vaccines and the majority of vaccines undergoing preclinical and clinical testing, employ a regimen of at least three doses (12, 18–22, 48, 49). Furthermore, the candidate vaccine prevented the development of carditis and lymphadenopathy that were observed in the control group and are consistent with LD infection (32–36). This lack of carditis and lymphadenopathy in vaccinated mice is in agreement with the qPCR data, which confirmed that *B. burgdorferi* was unable to disseminate to the heart tissue and lymph nodes of vaccinated mice.

In the United States, 1-10% of LD patients develop carditis (50). Lyme carditis may be caused by multiple factors, including direct infection of the myocardium, excessive macrophagic and lymphocytic infiltration, and autoimmunity (51). In humans, Lyme carditis most commonly affects the atrioventricular node (50, 51). In comparison, carditis in mice typically presents with inflammation of the connective tissue in the heart base and aorta (33). The ability of LD vaccines to prevent carditis associated with infection has not been well documented. In the present study, we observed mild-to-moderate mononuclear-neutrophilic inflammation of the heart base and aorta in all infected mice and, most importantly, we report the complete absence of carditis in all mice vaccinated with pVAX1-OspC:LNP. Overall, these findings demonstrate that vaccines targeting OspC alone are suitable for the prevention of infection with homologous strains of *B. burgdorferi.*

A major limitation of vaccines that target OspC exclusively is the genetic heterogeneity of the OspC antigen. Over 20 different subtypes of OspC have been found among *B. burgdorferi* pathogens and cross-protection between subtypes is limited (52). Prior infection with *B. burgdorferi* or vaccination with recombinant OspC protein has shown to protect against needle- or tick-challenge of bacterial strains only if they possess the homologous OspC allele (53–55). For the greatest biological relevance, human LD vaccines should confer protection against heterologous challenge using a tick-infection model. Therefore, future studies may explore the efficacy of DNA vaccines encoding multiple OspC subtypes. In addition, OspC may be used in combination with tick salivary proteins, such as Salp15, or other *B. burgdorferi* surface proteins to improve the breadth of protection. Future studies should therefore evaluate the efficacy of mono- and polyvalent vaccines in larger populations featuring both sexes.

Due to their ease of production and cost-efficiency, DNA-LNP platforms are ideal for the administration of multiple antigens. Polyvalent DNA-LNP vaccines can be administered in a multitude of ways, including: a) a single plasmid expressing multiple antigens, b) multiple plasmids each encoding an antigen and encapsulated in separate LNPs and c) multiple plasmids each encoding an antigen of interest and encapsulated together in an LNP. Additionally, LNPs encapsulating different plasmids can be formulated and injected together or separately. The optimal administration method of polyvalent DNA vaccines delivered by LNPs requires further investigation.

To our knowledge, few studies have demonstrated the efficacy of LNPs for the delivery of DNA vaccines. DNA vaccines delivered by syringe without a carrier are generally considered to have low immunogenicity and may require the addition of CpG or other adjuvants (56). Additionally, unformulated DNA vaccines are susceptible to DNase degradation. Several studies have demonstrated that encapsulation with LNPs protects the DNA payload against treatment with DNase enzymes (57–59). Furthermore, we demonstrate that delivery of DNA by LNPs induces sufficient immune responses without the incorporation of an adjuvant or use of a specialized immunization device. The DNA-LNP platform offers an advantage over conventional LD vaccine platforms, such as recombinant protein and bacterin vaccines, which also require the use of adjuvants. Together, the rapid production, ease of administration, and immunogenicity of DNA-LNP vaccines, render this platform an attractive candidate for LD vaccine development.

Overall, our study demonstrates the ability of LD vaccines targeting homologous OspC to confer sterile immunity in susceptible tissues and afford effective protection against Lyme carditis and lymphadenopathy. This study also provides support for the use of LNPs in the formulation of DNA vaccines in addition to their conventional use in mRNA vaccines. Given the increasing rates of LD in North America and the genetic diversity of the causative agent, the conclusions drawn from this study could benefit the generation and application of broadly protective human LD vaccines.

## Data availability statement

The original contributions presented in the study are included in the article/**Supplementary Material**. Further inquiries can be directed to the corresponding authors.

## Ethics statement

The animal study was reviewed and approved by the Health Canada Institutional Animal Care Committee.

## Author contributions

AP, SNTR, WZ, LT, JW, CG, and XL contributed to the conceptualization of this project. AP, CL JC, EL, DP, GF, and JG performed data generation and analysis. WC, GC, SS, MR-M, LW, MJWJ and XL assisted with funding acquisition, supervision, and project administration. AP, SNTR, WZ, and LT were involved in data interpretation and manuscript preparation. The first draft of the manuscript was written by AP and the final submission was approved by all authors.
