# Supplementary material for: DNA lipid nanoparticle vaccine targeting outer surface protein C affords protection against homologous Borrelia burgdorferi needle challenge in mice
Source: Front Immunol. 2023 Mar 16;14:1020134. doi: 10.3389/fimmu.2023.1020134 (PMC10060826; doi:10.3389/fimmu.2023.1020134)
Supplement: Supplementary file 1 [file DataSheet_1.docx]

Supplementary Material

# Supplementary Figures

#
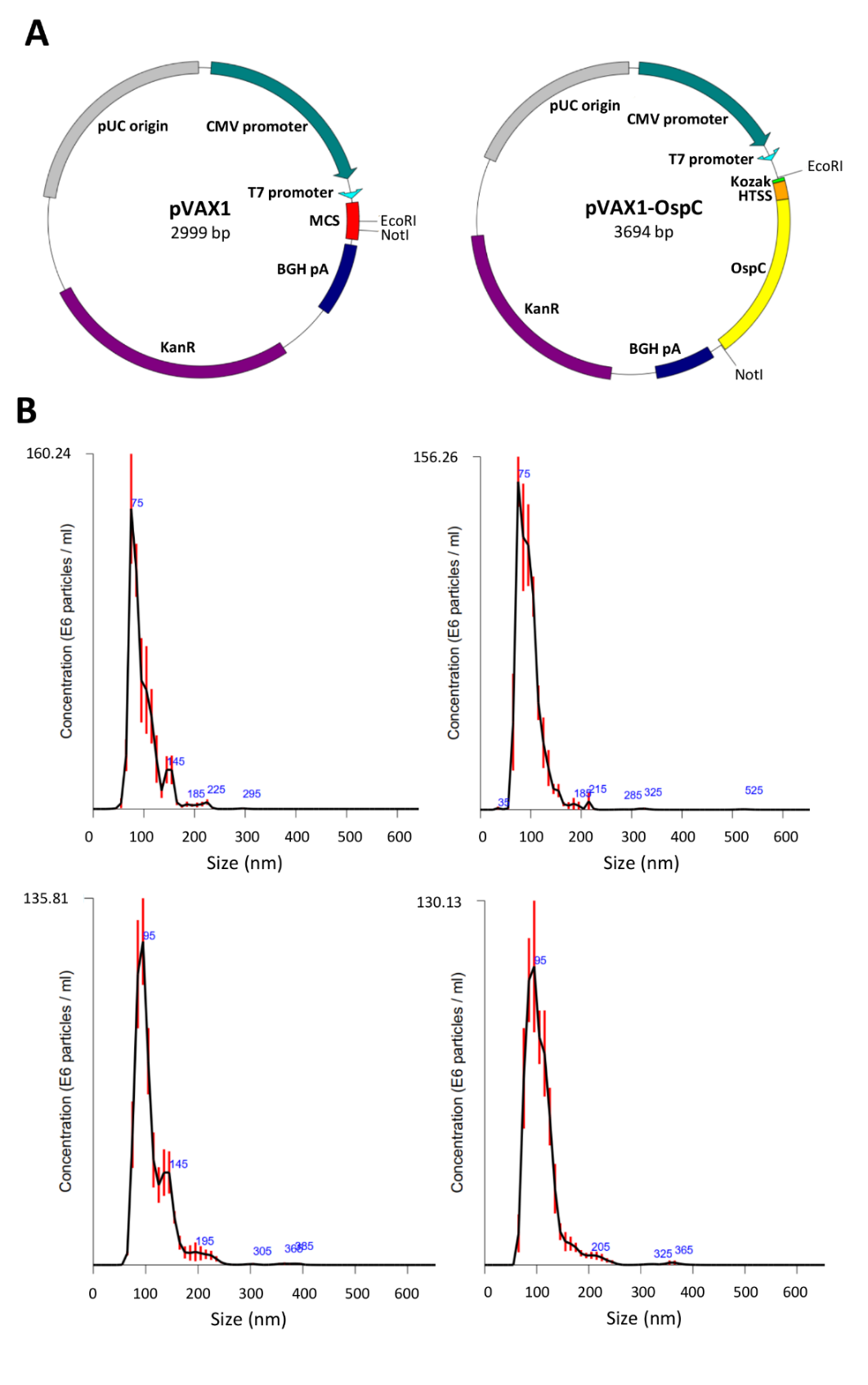


**Supplementary Figure 1.** DNA lipid nanoparticle vaccine design and characterization. **(A)** Schematics depicting the parental pVAX1 plasmid (pVAX1) and pVAX1 plasmid engineered to express outer surface protein C (pVAX1-OspC). **(B)** Size distributions of pVAX1:LNP (left) and pVAX1-OspC:LNP (right) formulations determined by nanoparticle tracking analysis. Top row: preparations for prime injections, bottom row: preparations for boost injections. Red error bars indicate standard error of the mean. CMV: cytomegalovirus, MCS: multiple cloning site, BGH pA: bovine growth hormone polyadenylation signal, KanR: kanamycin resistance gene, hTSS: human tyrosinase signal sequence.
